# Fatigue in major depression is linked to depression severity, not sleep duration or anterior cingulate cortex neurochemistry

**DOI:** 10.1016/j.jad.2025.121076

**Published:** 2026-01-02

**Authors:** Samir Batheja, Jie Yang, Yunhan Liao, Kenneth Wengler, Xiang He, Farzana Z. Ali, Russell Rozensky, Christine DeLorenzo

**Affiliations:** a Yale University, United States of America; b Stony Brook University, United States of America; c Mount Sinai, United States of America; d Northwell Health, United States of America; e UCLA, United States of America

**Keywords:** Depression, Sleep, Fatigue, GABA, Glx, Magnetic resonance spectroscopy

## Abstract

In major depressive disorder (MDD), it is unclear whether fatigue is a result of poor sleep or depression, and its biological basis is unknown. To explore this, Magnetic Resonance Spectroscopy (^1^H-MRS) in the anterior cingulate cortex (ACC) was acquired in unmedicated participants with MDD (*N* = 76). Gamma-aminobutyric acid (GABA+) and Glx (combination of glutamate and glutamine) concentrations were quantified from an average co-edited difference spectrum, with preprocessing using Gannet and spectral fitting using TARQUIN. Participants (median age: 23.5±14.2 years) completed sleep and depression questionnaires. The Wilcoxon rank sum or Kruskal-Wallis test was used to examine the marginal difference in continuous variables between levels of fatigue. Mediation effects of depression severity, GABA+, and Glx concentrations on the relationship between sleep duration and fatigue were evaluated using multivariable logistic regression models with fatigue as the dependent variable. Bayes Factor hypothesis testing examined the strength of evidence. Separate analyses were repeated with sleep efficiency as the main predictor. No significant relationship was observed between sleep duration/efficiency or GABA+/Glx and fatigue. Depression severity was significantly associated with fatigue and had a significant mediation effect on the relationship between sleep duration and fatigue. Specifically, for every additional hour of sleep, the odds of feeling fatigued nearly every day decreased by 11 % via an indirect mediating effect of decreased depression severity. GABA+/Glx did not mediate the effects of sleep on fatigue. The results suggest that, in this relatively young population, interventions to improve depression in MDD may be beneficial in reducing the debilitating effects of fatigue.

## 1. Introduction

The World Health Organization estimates that depression affects 280 million people globally (World Health Organization, 2023). Depression is associated with both improper sleep and fatigue. Related to the former, sleep disturbance is a criterion for the diagnosis of major depressive disorder (MDD) according to the Diagnostic and Statistical Manual of Mental Disorders (DSM, (Amercian Psychiatric Association, 2022), and about 90 % of depressed patients experience poor sleep quality (Jones et al., 1987; Tsuno et al., 2005). Related to the latter, fatigue, a feeling of low energy, diminished focus, and tiredness, requiring rest but not necessarily sleep (Shen et al., 2006), is also one of the most common symptoms of depression experienced by 73–97 % of depressed patients (Ferentinos et al., 2009; Harrington, 2012; Tylee et al., 1999). Fatigue can impair daily activities and exacerbate the symptoms of MDD (Carney et al., 2014; Fortier-Brochu et al., 2010), as well as increase the risk for MDD (Addington et al., 2001). In non-depressed individuals, sleep and fatigue are often coupled (Goldman et al., 2008; Veasey et al., 2002) and, in those with insomnia, fatigue is the most consistently reported daytime complaint (Fortier-Brochu et al., 2010). However, the feeling of fatigue may be more influenced by quality of life than sleep quality (Fortier-Brochu et al., 2010). In fact, across adults with poor sleep, fatigue is significantly higher in those exhibiting distress compared to poor sleepers without distress (Alapin et al., 2000). This raises the question of whether the feeling of fatigue is primarily related to poor sleep or depression severity within MDD. Knowing this can help optimize treatment of fatigue. Further, the biological basis of fatigue within MDD remains to be determined.

Glutamate and gamma-aminobutyric acid (GABA) are the primary excitatory and inhibitory neurotransmitters (Oa, 2002) and therefore contribute to arousal, fatigue, and sleep (Hansson and Rönnbäck, 2004; Nitz and Siegel, 1996; Riemann et al., 2010; Rönnbäck and Hansson, 2004). Increased glutamate or Glx (combined signal from glutamate and glutamine) levels have been observed in the brains of fatigued rats compared to controls (Li et al., 2020; Świątkiewicz et al., 2017). In human studies, an increase in cortical Glx following exercise-induced fatigue, as compared to pre-exercise values, was detected using proton magnetic resonance spectroscopy (^1^H-MRS) (Maddock et al., 2011). Higher Glx levels were also observed in people performing high-demand cognitive work who were fatigued compared to those with low-demand cognitive work (Wiehler et al., 2022). In chronic fatigue syndrome (CFS), a decreased level (Chen, 2017) or no differences (Kelly et al., 2009) in GABA have been reported in the anterior cingulate cortex (ACC). Although the mechanism of fatigue may be different in these cases compared to in depression, to our knowledge, no studies have examined the association between GABA and fatigue in healthy participants or the neurobiology of fatigue within MDD.

Within MDD, reports of glutamate and GABA levels are equivocal. In MDD patients *without* reported sleep disorders, region-specific decreased (Arnone et al., 2015; Benson et al., 2020; Hasler et al., 2007; Lener et al., 2017; Luykx et al., 2012; Moriguchi et al., 2019) and increased (Godlewska et al., 2017; Kantrowitz et al., 2021) glutamate or Glx was reported compared to non-depressed controls. However, in young adults with depression *and* late sleep midpoint or insomnia, higher Glx in the ACC (Naismith et al., 2014) and the parieto-occipital cortex (Meyerhoff et al., 2014) have been reported. Higher Glx in the dorsolateral prefrontal cortex has also been reported in insomnia patients compared to a control group (Spiegelhalder et al., 2016).

Similarly, in patients with MDD and *without* reported sleep disorders, region-specific reduced GABA (Alcaro et al., 2010; Northoff and Sibille, 2014; Sanacora et al., 2004; Schür et al., 2016) or no differences in GABA (Benson et al., 2020; Brennan et al., 2017; Godlewska et al., 2017; Kelly et al., 2009; Walter et al., 2009) have been reported compared to non-depressed controls. However, patients who have depression with insomnia or poor sleep quality compared to controls exhibit lower GABA concentrations in multiple brain regions (Bhagwagar et al., 2008; Goddard, 2016; Meyerhoff et al., 2014; Park et al., 2020; Plante et al., 2012a; Plante et al., 2012b; Rosso et al., 2014; Sanacora, 2010; Winkelman et al., 2008). Similarly, studies have found reductions in GABA levels in individuals with insomnia (Plante et al., 2012a; Winkelman et al., 2008) compared to those without insomnia.

Based on the above, higher glutamate/Glx levels are associated separately with fatigue, insomnia, and sleep disturbances within MDD. Lower GABA levels have been associated with insomnia and sleep disturbances within MDD, but have not been examined in fatigue. Studies examining glutamate/Glx and GABA in MDD alone have been equivocal, potentially due to varying levels of sleep/fatigue. The present study, therefore, seeks to precisely assess the relationship between fatigue and depression, sleep, and these neurotransmitters. Specifically, we assess whether depression severity and/or GABAergic and glutamatergic mechanisms mediate the relationship between sleep and fatigue. A mediation model is applied because, while evidence exists that glutamate/Glx, GABA, and depression severity are differentially associated with sleep and fatigue, it is unknown if these variables serve as intermediary mechanisms by which poor sleep influences fatigue.

Glx and GABA levels were measured by ^1^H-MRS. Though previous findings varied by region, the ACC was chosen as it is implicated both in MDD and sleep (Kantrowitz et al., 2021) and it plays a vital role in the pathophysiology of mood and anxiety disorders due to its connectivity and control of limbic, hypothalamic, and reward-linked areas (Stevens et al., 2011). Specifically, glutamatergic activity in the ACC has been associated with MDD symptoms, sleep disturbances, as well as response to sleep deprivation (Artigas, 2021; Bains and Abdijadid, 2022; Hefti et al., 2013; Mariani et al., 2021; Wolf et al., 2016). Further, patients with MDD exhibit abnormal resting-state functional connectivity in ACC subregions (Wu et al., 2022). We hypothesized that the severity of depression, along with Glx and GABA levels in the ACC, mediate the relationship between sleep and fatigue in MDD. Specifically, we propose that reduced sleep, combined with increased depressive symptoms, elevated Glx, and decreased GABA levels, would be associated with an increase in fatigue. The outcome of this study could shed light on whether targeting sleep disturbances may alleviate fatigue in MDD independently of changes in depression severity.

## 2. Methods

### 2.1. Participants

The Institutional Review Board of Stony Brook University approved this study. Participants were screened for eligibility by trained clinicians and clinical raters. All participants in this study were part of a larger study using simultaneous positron emission tomography (PET) and magnetic resonance imaging (MRI) in a randomized, double-blind clinical trial of escitalopram (Hill et al., 2021). PET, structural/diffusion MRI, and actigraphy results from that study have been previously published, as well as ^1^H-MRS results with respect to treatment outcomes (Ali et al., 2022; Dai et al., 2025; Narayan et al., 2022). None of these studies examined fatigue or sleep. The inclusion criteria included ability to understand and sign informed consent, age of 18 years or older, diagnosis of current MDD, and a score of 22 or higher on the Montgomery-Asberg Depression Rating Scale (MADRS (Montgomery and Asberg, 1979); 22 is considered moderate depression). The MADRS was used for inclusion/exclusion screening, while the 17-item Hamilton Depression Rating Scale was used to evaluate pretreatment depression severity to avoid any inflation of the MADRS that may occur at screening from affecting the evaluation of depression severity. Exclusion criteria included current efficacious antidepressant treatment (for the treatment trial), contraindications to escitalopram, including a previous failure of escitalopram therapy (for the treatment trial), electroconvulsive therapy (ECT) within six months, lifetime history of psychosis or current psychotic symptoms, past diagnosis of bipolar disorder, actively suicidal, high potential for excessive substance use during the study period, significant active physical illness, significant neurological deficits, or contradictions to MRI or PET (e.g., metal implants or pregnancy).

Within days of imaging, participants completed a self-report sleep questionnaire, a hybrid of several different surveys, including an abridged Pittsburgh Sleep Quality Index (PSQI (Buysse et al., 1989). Based on the abridged PSQI survey, examining habitual sleep over the past month, only component 3 (self-reported sleep in hours) and component 4 (sleep efficiency) could be calculated. Component 4 is the percentage of time spent asleep while in bed, calculated by dividing the self-reported amount of time spent asleep by the total self-reported amount of time in bed.

Because of the unequal number of participants across fatigue levels, when evaluated dichotomously, the two levels were defined as: Nearly every day (47 participants, 61.84 %) vs. Other (fatigue 3–4 times a week [13 participants], 1–2 times a week [13 participants], or very rarely [3 participants]; total = 29 participants, 38.16 %).

### 2.2. Imaging acquisition

All participants were medication-free for at least three weeks before imaging. MRI was performed simultaneously with positron emission tomography (PET) on a Siemens mMR scanner with a 12-channel head coil (Siemens, Erlangen, Germany). Anatomical T1-weighted 3D magnetization prepared rapid gradient echo (MPRAGE) images were acquired with the following parameters: repetition time/echo time/inversion time (TR/TE/TI) = 2300/3.24/900 ms, flip angle = 9°, matrix size = 256 × 256 × 224, 0.87 mm isotropic resolution, bandwidth = 220 Hz/pixel, and parallel imaging factor = 2.

^1^H-MRS data were acquired as in Narayan et al (Narayan et al., 2022) using the MEGA-PRESS sequence (Mescher et al., 1998) with the following parameters: voxel size = 20 × 30 × 30 mm^3^, TR/TE = 1500/80 ms, acquisition bandwidth = 1200 Hz; pulse placement edit-on/edit-off = 1.9/7.5 ppm, and number of excitations edit-on/edit-off = 96/96. A TE of 80 ms was used to achieve greater selectivity of the editing pulses (40 Hz as opposed to standard 60 Hz), suppress co-edited macromolecules, as well as enable longer editing RF pulses – two 25 ms editing pulses were incorporated – for J-editing with improved selectivity (Edden et al., 2012).

**Fig. 1.**
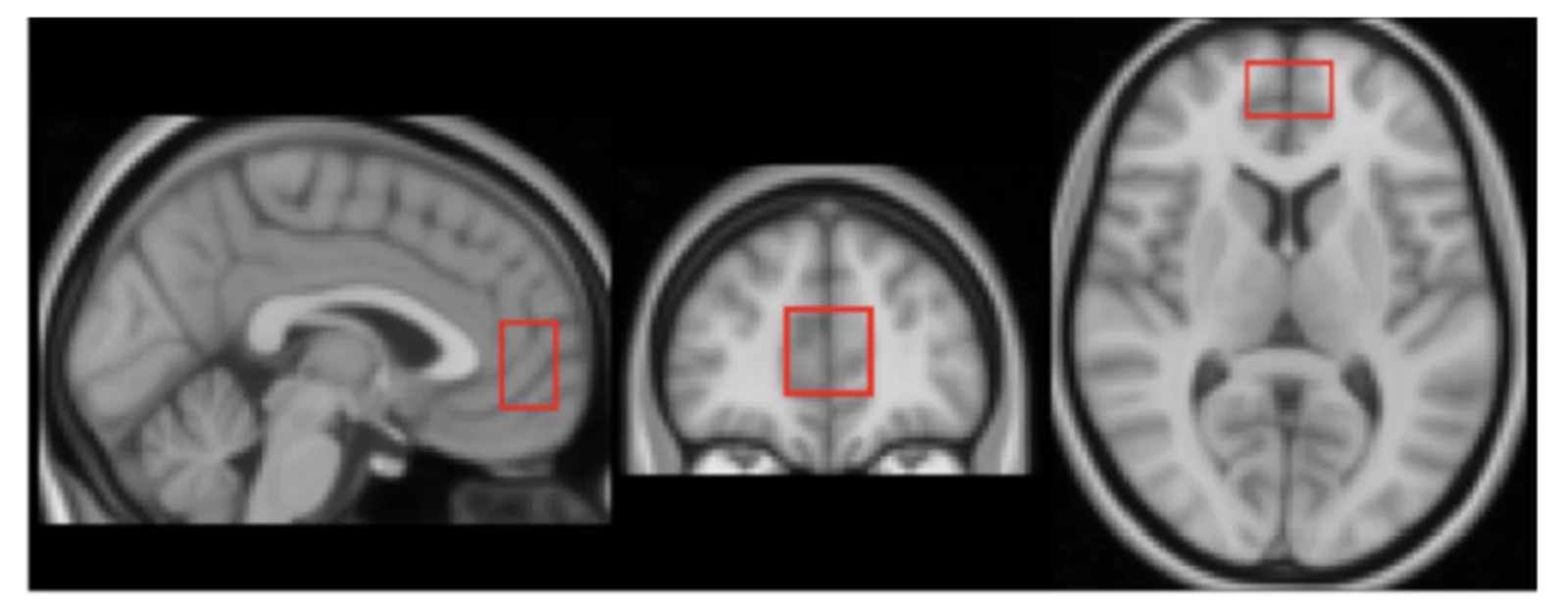
^1^H-MRS voxel size and location, shown in the sagittal (left), coronal (middle), and axial (right) views.

The single-voxel ^1^H-MRS acquisition was obtained in the midline ACC, manually positioned on each participant’s T1-weighted MPRAGE image and centered on the interhemispheric fissure, aligned to the curvature of the corpus callosum and positioned just superior to the genu. The voxel was oriented along the rostro–caudal axis of the ACC and adjusted to include predominantly dorsal/rostral ACC while avoiding contamination from adjacent cerebrospinal fluid by ensuring that the inferior boundary did not extend into the callosal sulcus and the posterior boundary did not encroach upon the lateral ventricles. Placement was performed by trained operators following a standardized protocol and verified in sagittal, coronal, and axial planes. A representative voxel placement in the ACC is shown in Fig. 1.

Two GABA-edited MEGA-PRESS acquisitions were collected and interleaved with a water-unsuppressed reference scan. The reference scan was acquired with the same parameters, except water suppression pulses were not applied, and the number of excitations = 16. Due to co-edited macromolecule (MM) contamination, GABA+ (i.e., GABA with MM) was assessed.

### 2.3. ^1^H-MRS data processing and quantification

Similar to Narayan et al (Narayan et al., 2022) and in line with best practices, visual inspection of all spectra was used as quality control (Kreis, 2016; Landheer and Juchem, 2021; Lin et al., 2021), as support for quantitative cutoffs such as the Crámer Rao Lower Bound (CRLB) as a basis for exclusion is equivocal, and widely used thresholds may cause bias in estimated mean concentrations (Kreis et al., 1995; Landheer and Juchem, 2021). Exclusions were due to field inhomogeneity within the ^1^H-MRS voxel as a result of either poor shimming or participant motion, with the latter causing subtraction errors that result in model-fitting failure. Inclusion of scans with motion artifact results in degraded spectral quality and can have further implications such as a reduced signal to noise ratio, lipid contamination, baseline fluctuation, insufficient water suppression, and incorrect quantification (Saleh et al., 2020).

^1^H-MRS processing was conducted in MATLAB (MathWorks Inc., Natick, MA) using Gannet, which allows preprocessing of individual spectra (Edden et al., 2014). Processing steps consisted of 1. coil combination and prephasing based on the reference scan; 2. zero-filling of data to obtain a nominal spectral resolution of 0.061 Hz/point; 3. eddy current correction (Klose, 1990); 4. line broadening of 3 Hz; 5. frequency and phase correction using the spectral registration method (Near et al., 2015) with correction parameters estimated from the edit-off data; 6. Difference spectrum estimation by pairwise subtraction of the edit-off data from the edit-on data. The two GABA-edited MEGA-PRESS acquisitions were processed independently and then averaged for a resulting difference spectrum generated from 192 edit-on and 192 edit-off excitations. The GABA+ and Glx concentrations were then quantified from the average co-edited difference spectrum using TARQUIN (Wilson et al., 2011), shown to provide more robust estimates of GABA+ compared to Gannet (Zöllner et al., 2021), with the average processed water unsuppressed reference scan used to estimate concentrations relative to water. A water concentration of 35,880 mM was assumed for brain tissue. Relaxation correction was applied using a water T_2_ of 83 ms and a metabolite T_2_ of 200 ms. No T_1_ relaxation correction was performed. The Kaiser basis set (Kaiser et al., 2008) was used. No tissue correction was performed.

While glutamine is present, it is believed that the Glx signal in the co-edited difference spectrum is predominantly glutamate due to reduced editing efficiency for glutamine compared to glutamate (van Veenendaal et al., 2018).

### 2.4. Statistical analysis

A chi-square test with exact *p*-values based on Monte Carlo simulation was utilized to examine the marginal association between categorical variables and fatigue (two levels: Nearly every day vs Other, and three levels: grouping “1-2 times a week” and “very rarely” together). The Wilcoxon rank sum or Kruskal-Wallis test was utilized to examine the marginal difference in continuous variables between participants with different levels of fatigue. Logistic regression models with and without each of depression severity, GABA+, and Glx were used to evaluate the relationship between sleep duration or sleep efficiency and fatigue, adjusting for sex and age. An odds ratio (OR) >1 indicated a higher risk of having fatigue nearly every day, while an OR < 1 indicated a lower risk. Mediation effects of depression severity, GABA+, and Glx on the effect of sleep duration or sleep efficiency on fatigue, while adjusting for age and sex, were tested individually. In contrast to a hierarchical regression approach, mediation analysis decomposes the total effect of an exposure into direct and indirect components.

The Odds Ratio Total Effect describes the overall association between an independent variable and a dependent variable encompassing all pathways, both direct and indirect. In mediation analysis, the odds ratio controlled direct effect (CDE) measures the effect of the independent variable on the dependent variable when the mediator is controlled at the mean level. The odds ratio natural direct effect (NDE) represents the effect of the independent variable on the dependent variable that occurs in the absence of the mediator’s effect. The odds ratio natural indirect effect (NIE) represents the effect of the independent variable on the dependent variable that operates through the mediator. A value in OR NIE significantly different from 1 should be expected if the mediation effect exists (Vanderweele and Vansteelandt, 2010). An OR NIE >1 indicates a higher risk of having fatigue nearly every day for an additional unit of sleep duration or sleep efficiency via the mediating effect of the mediator, while an OR NIE <1 indicates a lower risk of having fatigue nearly every day via the mediating effect of the mediator.

For all regression models that included sleep duration or sleep efficiency, the Bayes Factor (BF) was approximated using the Bayesian Information Criterion (BIC) of the null/reduced model and the BIC of the alternative/full model (Boykin et al., 2023). Bayes Factors complement traditional significance testing by quantifying the strength of evidence for associations. BF <1/3 and < 1/10 show moderate and strong evidence for the null hypothesis, respectively, while BF values between 1/3 and 3 indicate insufficient evidence to draw a conclusion for or against either hypothesis, and BF >3 and > 10 show moderate and strong evidence for the alternative hypothesis, respectively (Keysers et al., 2020).

Analyses were performed using SAS 9.4 (SAS Institute Inc., Cary, NC), and the significance level was set at 0.05.

## 3. Results

### 3.1. Participants

A total of 85 participants who met the DSM-IV criteria for current MDD were recruited by advertising and imaged. Nine participants were excluded due to incomplete sleep duration or fatigue information. Of the remaining 76 participants (median age: 23.5±14.2 years, 54 female), six had unreliable (>100 %) sleep efficiency values, and five had unreliable ^1^H-MRS imaging, and were only used in analyses where valid data were available. Participant information is shown in Table 1.

### 3.2. Relationship between sleep duration/efficiency and fatigue

**Fig. 2.**
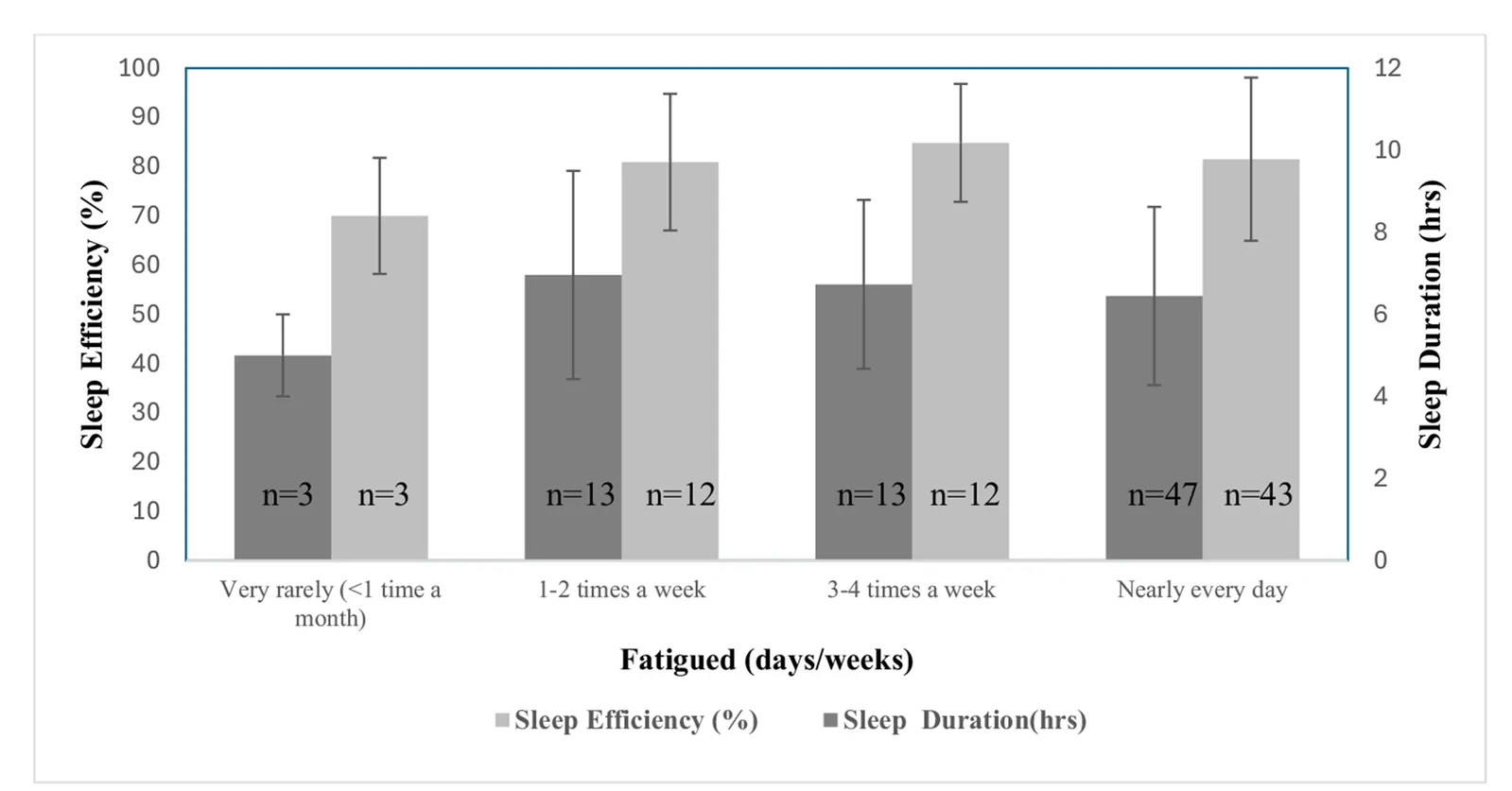
Bar graph showing sleep duration and efficiency by fatigue level. Data are expressed as average ± standard deviation. The number of participants is listed for each category.

As Table 1 shows, no significant difference was found between self-reported sleep duration or efficiency in the two fatigue levels. Even when examined in three levels (grouping “1-2 times a week” and “very rarely”), no significant relationship was found between self-reported sleep duration (*p*-value = 0.77) or efficiency (*p*-value = 0.48) and fatigue. Fig. 2 further depicts sleep duration and efficiency by all self-reported fatigue levels.

In addition, no significant relationship was found between self-reported sleep duration (Table 2) or efficiency (Table 3) and fatigue after adjusting for sex and age, without and with each of the three possible mediators (depression severity, GABA+, Glx). Sex was a significant predictor in both models when depression severity, but not GABA+ or Glx, was included. In the sleep duration model, female participants had 3.74 times the odds of reporting daily fatigue compared to males (Table 2, OR = 3.74, 95 % CI: 1.20–11.67, *p*-value = 0.02). Similarly, in the sleep efficiency model, the odds of reporting fatigue were also significantly higher for females (Table 3, OR = 3.62, 95 % CI: 1.05–12.43, *p*-value = 0.04). However, all Bayes Factors for models examining sleep duration/efficiency and fatigue were less than 1/3, indicating moderate evidence of a null hypothesis (sleep duration/efficiency had no relationship with fatigue).

### 3.3. Does depression severity, GABA+, or Glx mediate the relationship between sleep duration/efficiency and fatigue?

In the mediation analysis, the indirect effects of sleep duration on fatigue were mediated via depression severity (Table 4, OR NIE estimate = 0.89, 95 % CI: 0.78–0.99, *p*-value = 0.03) but not GABA+ or Glx. Direct effects were not detected (Table 4). For every additional hour of sleep duration, the odds of feeling fatigued nearly every day decreased by 11 % (estimated OR was 0.89) via *the indirect mediating effect of decreased depression severity*. The percentage mediated could not be accurately assessed due to the insignificant direct effect.

The mediation effect of depression severity did not reach significance when using sleep efficiency as the independent variable (Table 5, OR NIE estimate = 0.99, 95 % CI: 0.97–1.00, *p*-value = 0.13).

**Fig. 3.**
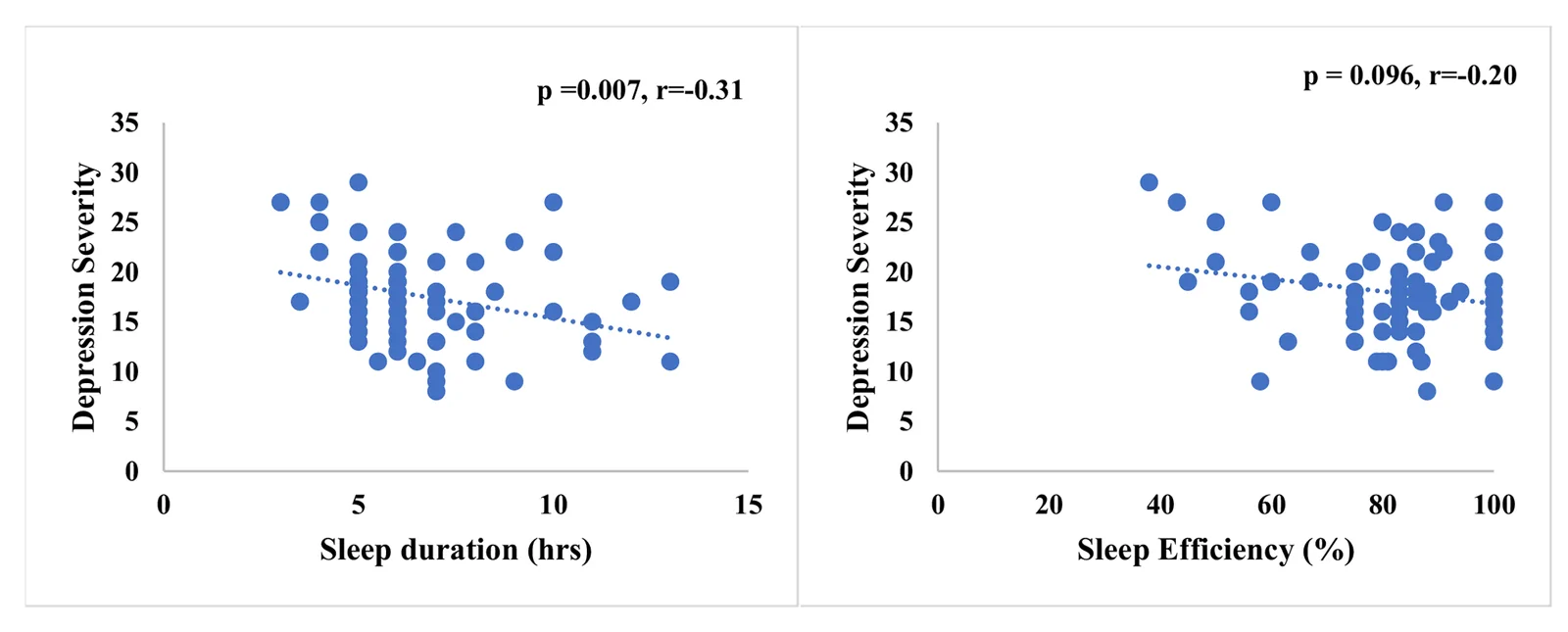
Depression Severity vs Sleep Duration (left) and Efficiency (right). The dotted line represents the best linear fit.

Given the mediation effects of depression in Table 4, to further explore the relationship between sleep duration, depression severity and fatigue, a multiple linear regression model regressing depression severity on sleep duration and a multivariable logistic regression model regressing fatigue on depression severity were run with an adjustment for sex and age. In the multiple linear regression model, sleep duration was significantly associated with depression severity (Estimated coefficient = −0.68, 95 % CI: −1.16 - −0.21, *p*-value <0.01), as participants with longer sleep duration had lower depression severity scores. The Bayes Factor for sleep duration was >10 (31.30), indicating strong evidence of the alternative hypothesis (sleep duration was significantly associated with depression severity). Consistently, Fig. 3 shows a significant inverse correlation between sleep duration and depression severity (Pearson correlation coefficient = −0.31, *p*-value <0.01, not observed with sleep efficiency, Pearson correlation coefficient = −0.20, *p*-value = 0.096).

In the multivariable logistic regression model, depression severity was also significantly associated with fatigue (estimated odds ratio = 1.18, 95 % CI: 1.04–1.33, *p*-value <0.01) after adjusting for sex and age. Participants with higher depression severity had a higher risk of feeling fatigued nearly every day, and the Bayes Factor for depression severity was >3 (Bayes Factor = 7.08, indicating moderate evidence of the alternative hypothesis -depression severity was significantly associated with fatigue).

**Fig. 4.**
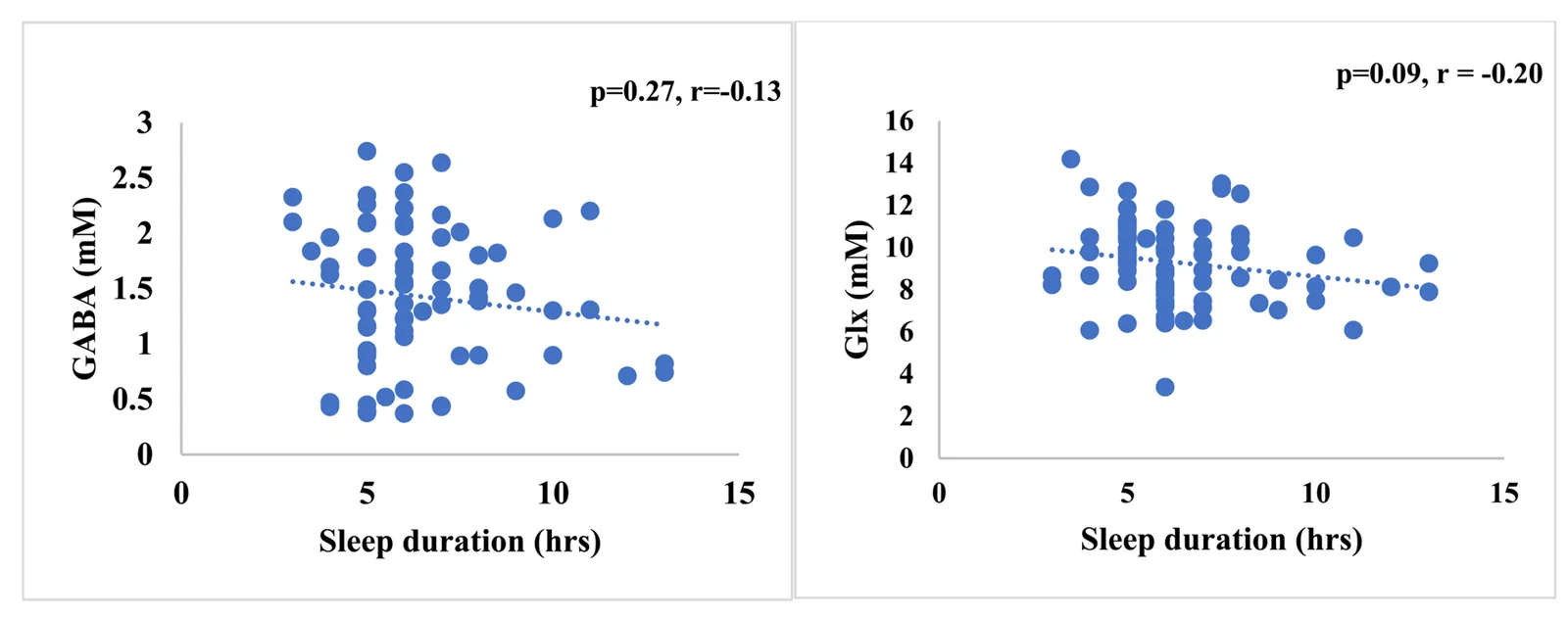
Gamma-aminobutyric acid (GABA, including macromolecules, MM) (left) and Glx (a combination of glutamate and glutamine) (right) vs. sleep duration. The dotted line represents the best linear fit.

For completeness, the relationship between sleep duration and GABA+/Glx was examined, and no significant correlations were observed (Fig. 4; GABA+, Pearson correlation coefficient = −0.13, *p*-value = 0.27, and Glx (Pearson correlation coefficient = −0.20, *p*-value =0.091).

## 4. Discussion

Previous studies have shown that depressed patients suffer from greater levels of fatigue than those with other diseases, such as cancer, or non-depressed controls (Anderson et al., 2003). Consistent with this finding, in the present study, >60 % of the depressed participants reported fatigue nearly daily. Yet, fatigue in depression is not well understood. Specifically, it is unclear whether fatigue in depression is a result of poor sleep, depression severity, or a separate mechanism. Therefore, in this study, we aimed to examine the relationship between sleep, fatigue, and depression severity.

We observed no significant relationship between sleep duration or efficiency and fatigue after adjusting for sex and age (with moderate evidence of this null finding, using the Bayes Factor) – suggesting that fatigue in MDD may not be entirely explained by the total hours one sleeps or their sleep quality. This is in opposition to what has been reported in those without depression, in which sleep and fatigue are often coupled (Goldman et al., 2008; Lavidor et al., 2003).

Although no direct association was observed between sleep and fatigue, mediation analysis suggests that the effect of sleep duration on fatigue was mediated by depression severity after controlling for age and sex (Table 4). This is similar to a previous study showing that the relationship between sleep duration and cognitive impairment was mediated by depression severity (Wang et al., 2023). In our study, although sleep duration did not directly affect the risk of feeling fatigued nearly every day (non-significant Odds Ratio Natural Direct Effect) and there was no direct association between sleep duration and fatigue, for every additional hour of sleep duration, the odds of feeling fatigued nearly every day decreased by 11 % via the indirect mediating effect of decreased depression severity (Odds Ratio Natural Indirect Effect). A multiple linear regression model and multivariable logistic regression model further illustrated that depression severity was significantly associated with sleep duration and fatigue.

Taken together, the results here suggest that MDD-associated sleep impairment is not the direct cause of MDD-associated fatigue (as sleep duration/efficiency was not significantly associated with fatigue). The significant Natural Indirect Effect combined with a non-significant Total Effect suggests that sleep duration does influence fatigue indirectly through depression severity. Yet, this indirect effect is counterbalanced or masked by the non-significant direct effect, resulting in a non-significant total effect. This could have treatment implications, as it implies that improving sleep in depression may not directly improve levels of fatigue. This fact, combined with the significant association between depression severity and both sleep duration and fatigue, as well as the mediating effects of depression, suggests that MDD-related biology could underly all three variables.

Findings also suggest that sex may play a role in fatigue in MDD. Sex became a significant covariate in sleep duration (Table 2) and sleep efficiency (Table 3) models when depression severity was included. This suggests that the relationship between sex and fatigue may be unmasked or amplified when adjusting for depression severity, pointing to potential sex-specific pathways by which depression contributes to fatigue. Consistent with this finding, prior research has noted that women with depression are more likely to report somatic symptoms, including fatigue (Dzurec et al., 2002; Yang and Han, 1997). Interestingly, the effect of sex on fatigue became non-significant when GABA+ or Glx were included in the models. This attenuation may be due to overlapping variance between sex and these metabolites, possibly reflecting underlying neurobiological differences between males and females in GABAergic or glutamatergic signaling.

The results of the regression models, mediation models, and correlation analysis suggest that GABA+ / Glx levels in the ACC are not significantly associated with either sleep duration/efficiency or fatigue and that these levels do not mediate the relationship between sleep and fatigue. This contrasts with previous studies examining fatigue in response to exercise or high-demand cognitive work and in CFS with poor sleep quality. These could reflect a different mechanism than depression-associated fatigue.

Limitations: Fatigue is a subjective symptom which, in this study, was measured by self-report using a question from the PSQI questionnaire. Other scales may provide more sensitivity. For example, the fatigue severity scale (FSS), a measurement scale based on a self-report questionnaire, is commonly used to measure fatigue (Valko et al., 2008), but was not acquired in this study. Similarly, in this study, sleep duration and efficiency were provided by self-report (or calculated from selfreported data in the case of sleep efficiency). Although depression severity could influence impressions of sleep levels, the mediation model suggests that sleep duration and depression severity did not have the same association with fatigue. As shown in Fig. 2, however, there were numerous reports of 100 % sleep efficiency, which is unlikely to be accurate. This may be one reason why the associations and mediation effects were significant, using the more reliable sleep duration rather than sleep efficiency.

Further, as GABA was quantified using MEGA-PRESS in this work, it yields a composite GABA+ signal that includes macromolecule (MM) contamination that may exhibit interindividual variation. While not a pure measure of GABA, it remains a widely used and accessible method in clinical research. However, GABA+ quantification using the mMR rather than an MRI-only scanner does result in a reduction of quality of spectra, requiring rigorous quality control. Additionally, the use of Glx may have obscured findings by not considering glutamate and glutamine separately.

Finally, Glx and GABA+ were only measured in the ACC. Metabolite levels in other brain regions, for example, the lateral prefrontal cortex, may have a better association with fatigue (Wiehler et al., 2022). It is also possible that other neurotransmitters mediate the relationship between sleep and fatigue. For example, it was reported that serotonin, norepinephrine, dopamine, and histamine, which are implicated in both MDD and antidepressant mechanisms (Demyttenaere et al., 2005; Stahl et al., 2003), may underlie symptoms of fatigue in MDD (Demyttenaere et al., 2005).

The strength of this study is the large number of participants (>70) with careful assessment of depression (Hamilton Depression Rating Scale used as a measure of depression severity, separate from the MADRS used for inclusion/exclusion), use of brain imaging, and the rigorous statistical analysis examining brain imaging and self-report data. To our knowledge, this may be the most extensive study examining the relationship between fatigue and depression in people with MDD.

## 5. Conclusions

To our knowledge, this is the first study to examine GABA+ and Glx in the ACC in relation to depression, fatigue, and sleep. Unlike previous reports in non-depressed controls, neither sleep nor fatigue was significantly associated with GABA+ or Glx in the ACC in this cohort of depressed participants. This suggests that the relationship between brain metabolites and sleep/fatigue may be more complex in those with MDD and warrants further study. Importantly, results suggest that depression severity is a mediator accounting for the association between fatigue and sleep, suggesting a potential for depression-targeting interventions to mitigate fatigue symptoms.

## Figures and Tables

**Table 1:** Participant information. “Other” is feeling fatigue 3–4 times a week, 1–2 times a week, or very rarely. Depression severity is quantified from the Hamilton Depression Rating Scale. GABA+ and Glx (a combination of glutamate and glutamine) were quantified from Magnetic Resonance Spectroscopy (^1^H-MRS).

Variable		Total	Fatigue nearly everyday	n	Other	n	p-Value*
Age		23.45 ± 14.15	24.10 ± 16.40	47	22.80 ± 12.30	29	0.82
Depression severity		17.50 ± 5.50	18.00 ± 6.00	47	16.00 ± 5.00	29	<0.01
GABA+ (mM)		1.42 ± 1.07	1.37 ± 0.91	44	1.66 ± 1.27	27	0.55
Glx (mM)		9.35 ± 2.46	9.41 ± 2.33	44	9.35 ± 3.34	27	0.85
Sleep duration (hours)		6.00 ± 2.25	6.00 ± 2.50	47	6.00 ± 2.00	29	0.56
Sleep efficiency (%)		83.00 ± 15.00	86.00 ± 16.00	43	83.00 ± 13.00	27	0.61
Sex	Female	54 (71.05 %)	78.72 %	37	58.62 %	17	0.06
	Male	22 (28.95 %)	21.28 %	10	41.38 %	12	

Note: For categorical variables, row percentages were reported; for continuous variables, median+/− interquartile range (IQR) were reported.

* : For continuous variables, *p*-values were based on the Wilcoxon rank sum test; for categorical variables, *p*-values were based on the Chi-squared test with exact *p*-value from Monte Carlo simulation.

**Table 2:** Relationship between sleep duration and fatigue. Estimated odds ratio (OR) and its 95 % confidence interval (CI) for the relationship between sleep duration and fatigue based on logistic regression models. GABA+ and Glx (a combination of glutamate and glutamine) were quantified from Magnetic Resonance Spectroscopy (^1^H-MRS).

Effect	OR (95 % CI)	p-Value*	Bayes factor for sleep duration
**Without mediator effect**			
Sleep duration	0.97 (0.78, 1.21)	0.81	0.12
Age	1.01 (0.97, 1.04)	0.68	
Sex	2.63 (0.94, 7.36)	0.06	
**Including depression severity**			
Sleep duration	1.10 (0.85, 1.42)	0.46	0.15
Age	1.01 (0.97, 1.04)	0.73	
Sex	3.74 (1.20, 11.67)	0.02	
Depression severity	1.20 (1.05, 1.36)	<0.01	
**Including GABA+**			
Sleep duration	0.93 (0.75, 1.17)	0.56	0.14
Age	1.00 (0.97, 1.04)	0.82	
Sex	2.31 (0.80, 6.66)	0.12	
GABA+	0.75 (0.34, 1.66)	0.48	
**Including Glx**			
Sleep duration	0.93 (0.74, 1.17)	0.54	0.14
Age	1.00 (0.96, 1.04)	0.95	
Sex	2.45 (0.84, 7.15)	0.10	
Glx	0.91 (0.70, 1.19)	0.50	

* : *p*-values were based on type3 test from multivariable logistic regression models.

**Table 3:** Relationship between sleep efficiency and fatigue. Estimated odds ratio (OR) and its 95 % confidence interval (CI) for the relationship between sleep efficiency and fatigue based on logistic regression models. GABA+ and Glx (a combination of glutamate and glutamine) were quantified from Magnetic Resonance Spectroscopy (^1^H-MRS).

Effect	OR (95 % CI)	p-Value*	Bayes factor for sleep efficiency
**Without mediator effect**			
Sleep efficiency	1.00 (0.97, 1.03)	0.96	0.12
Age	1.01 (0.97, 1.05)	0.57	
Sex	2.29 (0.78, 6.75)	0.13	
**Including depression severity**			
Sleep efficiency	1.01 (0.97, 1.05)	0.56	0.14
Age	1.01 (0.97, 1.05)	0.53	
Sex	3.62 (1.05, 12.43)	0.04	
Depression severity	1.22 (1.07, 1.39)	<0.01	
**Including GABA+**			
Sleep efficiency	1.00 (0.97, 1.04)	0.94	0.12
Age	1.01 (0.97, 1.04)	0.70	
Sex	2.33 (0.78, 6.99)	0.13	
GABA+	0.79 (0.34, 1.80)	0.57	
**Including Glx**			
Sleep efficiency	1.00 (0.97, 1.04)	0.82	0.13
Age	1.01 (0.97, 1.05)	0.73	
Sex	2.33 (0.77, 7.05)	0.13	
Glx	0.97 (0.74, 1.27)	0.82	

* : *p*-values were based on type3 test from multivariable logistic regression

**Table 4:** Mediators of the relationship between sleep duration and fatigue. Mediation effect test for each possible mediator for the relationship between sleep duration and fatigue based on the generalized linear regression models.

Effect	Estimated values (95 % CI)*	p-Value
**Including depression severity**		
Odds Ratio Total Effect	0.97 (0.73, 1.22)	0.84
Odds Ratio Controlled Direct Effect (CDE)	1.10 (0.82, 1.38)	0.48
Odds Ratio Natural Direct Effect (NDE)	1.10 (0.82, 1.38)	0.48
Odds Ratio Natural Indirect Effect (NIE)	0.89 (0.78, 0.99)	0.03
**Including GABA+**		
Odds Ratio Total Effect	0.95 (0.73, 1.16)	0.61
Odds Ratio Controlled Direct Effect (CDE)	0.93 (0.72, 1.15)	0.55
Odds Ratio Natural Direct Effect (NDE)	0.93 (0.72, 1.15)	0.55
Odds Ratio Natural Indirect Effect (NIE)	1.01 (0.97, 1.05)	0.55
**Including Glx**		
Odds Ratio Total Effect	0.95 (0.73, 1.16)	0.61
Odds Ratio Controlled Direct Effect (CDE)	0.93 (0.72, 1.14)	0.52
Odds Ratio Natural Direct Effect (NDE)	0.93 (0.72, 1.14)	0.52
Odds Ratio Natural Indirect Effect (NIE)	1.02 (0.97, 1.07)	0.53

* : Confidence intervals were from Wald Confidence Intervals.

**Table 5:** Mediators of the relationship between sleep efficiency and fatigue. Mediation effect test for each possible mediator for the relationship between sleep efficiency and fatigue based on generalized linear regression models.

Effect	Estimated values (95 % CI)*	p-Value
**Including depression severity**		
Odds Ratio Total Effect	1.00 (0.96, 1.04)	0.96
Odds Ratio Controlled Direct Effect (CDE)	1.01 (0.97, 1.05)	0.56
Odds Ratio Natural Direct Effect (NDE)	1.01 (0.97, 1.05)	0.56
Odds Ratio Natural Indirect Effect (NIE)	0.99 (0.97, 1.00)	0.13
**Including GABA+**		
Odds Ratio Total Effect	1.00 (0.97, 1.04)	0.83
Odds Ratio Controlled Direct Effect (CDE)	1.00 (0.97, 1.04)	0.94
Odds Ratio Natural Direct Effect (NDE)	1.00 (0.97, 1.04)	0.94
Odds Ratio Natural Indirect Effect (NIE)	1.00 (0.99, 1.01)	0.59
**Including Glx**		
Odds Ratio Total Effect	1.00 (0.97, 1.04)	0.84
Odds Ratio Controlled Direct Effect (CDE)	1.00 (0.97, 1.04)	0.82
Odds Ratio Natural Direct Effect (NDE)	1.00 (0.97, 1.04)	0.82
Odds Ratio Natural Indirect Effect (NIE)	1.00 (1.00, 1.00)	0.83

* : Confidence Intervals were from Wald Confidence Intervals.
